# Histological Insights into Testicular Apoptosis Associated with Spermatogenesis in Pre-Pubertal and Adult Meagre (*Argyrosomus regius*)

**DOI:** 10.3390/ani15182668

**Published:** 2025-09-11

**Authors:** Gianluca Ventriglia, Neil Duncan, Ignacio Giménez, Constantinos C. Mylonas, Chrysovalentinos Pousis, Caterina Varvara, Luisa Valentini, Letizia Passantino, Aldo Corriero, Rosa Zupa

**Affiliations:** 1 Department of Veterinary Medicine, University of Bari Aldo Moro, 70010 Valenzano, Italy; gianluca.ventriglia@uniba.it (G.V.); caterina.varvara96@gmail.com (C.V.);; 2 Institute of Agrifood Research and Technology, 43540 La Ràpita, Spain; 3 Rara Avis Biotec, S. L., 46002 Valencia, Spain; 4 Institute of Marine Biology, Biotechnology and Aquaculture, Hellenic Center for Marine Research, 71003 Heraklion, Greece; mylonas@hcmr.gr; 5 DiMePRe-J, University of Bari Aldo Moro, 70010 Valenzano, Italy; luisa.valentini@uniba.it (L.V.); letizia.passantino@uniba.it (L.P.)

**Keywords:** fish, testis, gametogenesis, recombinant follicle stimulating hormone, seasonal reproduction

## Abstract

To overcome reproductive dysfunctions, recombinant gonadotropin hormones have been synthesised in different fish species. We were interested in gaining further insight into the effects of recombinant follicle-stimulating hormone (rFsh) in meagre *Argyrosomus regius* spermatogenesis and in improving the existing knowledge on the role of testicular apoptosis. Germ cell composition and testicular apoptosis were compared in pre-pubertal fish treated with rFsh (PreP-Fsh) and controls (PreP-C), and adult meagre with testes in the proliferative (Adult-Pro) and meiotic (Adult-Meio) spermatogenesis phases. A lower density of committed spermatogonia was observed in PreP-Fsh compared with PreP-C and in Adult-Meio compared with Adult-Pro. In pre-pubertal fish and in Adult-Pro, apoptosis affected mainly individual germ cells and Sertoli cells. In Adult-Meio, a high number of cysts containing apoptotic germ cells was observed, and apoptosis often involved entire clones of germ cells. rFsh reduced testicular apoptosis in pre-pubertal fish, whereas the advancement of spermatogenesis in adults was associated with an increased density of apoptotic cells. The present study corroborated previous observations on the effects of rFsh on spermatogenesis in pre-pubertal meagre and provided further insights on the changing role of apoptosis in the pre-pubertal phase versus different phases of spermatogenesis.

## Simple Summary

Recombinant gonadotropins—synthetic versions of pituitary hormones—are increasingly utilised in aquaculture for broodstock management. In this study, we explored how recombinant follicle-stimulating hormone (rFsh) affects spermatogenesis in meagre (*Argyrosomus regius*), with the aim of enhancing our understanding of testicular apoptosis—a common form of programmed cell death involved in modulating germ cell development and sperm output. We assessed germ cell composition and testicular apoptosis in both pre-pubertal meagre treated with rFsh and adult fish in two spermatogenic phases (proliferative and meiotic). In pre-pubertal fish, rFsh treatment led to larger testes, wider seminiferous tubules, and increased sperm presence. There was a reduction in early spermatogonia (undifferentiated germ cells) but a rise in dividing germ cells, indicating progression toward meiosis. Notably, treated fish exhibited significantly lower spermatogonial apoptosis compared to controls, suggesting rFsh promotes germ cell survival by mitigating natural cell death. In adult fish, the density of spermatogonia decreased with the progression of spermatogenesis, whereas apoptosis increased and often involved entire clones of germ cells. Overall, the study demonstrates that rFsh effectively induces testicular maturation in pre-pubertal meagre by promoting progression toward meiosis and highlights how the role of apoptosis varies across reproductive stages.

## 1. Introduction

The meagre *Argyrosomus regius* (Asso, 1801) is a member of the Sciaenidae family, native to the eastern Atlantic, including the Mediterranean and the Black Sea [1]. It is a valued aquaculture species whose production in the EU is gradually increasing [2,3]. Meagre broodstocks reared in tanks do not reproduce spontaneously due to a reproductive dysfunction that, in females, prevents oocytes from undergoing maturation after the completion of vitellogenesis, and, in males, results in the production of low-quality milt [4]. This dysfunction is commonly alleviated by treatment with gonadotropin-releasing hormone agonists (GnRHa) [2,4,5,6], either alone or in combination with thermal/photothermal control [7]. Meagre reach sexual maturity at 3–4 years of age [2,8], and attempts are being made to advance puberty in order to reduce the generation time in selective breeding programmes, thus speeding up the production of fish with improved characteristics, such as faster growth, more efficient feed consumption, increased disease resistance, etc. To this end, meagre recombinant follicle-stimulating hormone (rFsh) and recombinant luteinizing hormone (rLh) were synthesised and administered to 18-month-old pre-pubertal males, which showed signs of spermatogenesis but without producing releasable milt [9,10]. The rFsh treatment induced an increase in testicular mass, seminiferous tubule size, and the number of luminal spermatozoa. Moreover, rFsh-treated meagre had a lower density of proliferating single type A spermatogonia, a higher density of spermatocysts containing committed spermatogonia or primary spermatocytes, and a lower incidence of testicular apoptosis compared to the control pre-pubertal fish [9]. The high level of germ cell apoptosis and its reduction after rFsh administration suggested that the elevation of plasma Fsh/sex steroids was still insufficient to support the finalisation of the spermatogenesis process during the pre-pubertal phase, and the committed germ cells that could not proceed in the spermatogenesis process died by apoptosis.

The role of apoptosis in teleost fish spermatogenesis is not fully understood. In amniote vertebrates, the testis is composed of a fixed number of “immortal” Sertoli cells [11] that support successive waves of spermatogenesis, and apoptosis plays an important role in maintaining the correct germ cell/Sertoli cell ratio and in eliminating aberrant germ cells [12,13]. In anamniote vertebrates, Sertoli cells proliferate in parallel with germ cells [11,14,15], and a mechanism to maintain a balance between Sertoli and germ cells may then not be required. In teleost fish, as well as in other seasonally breeding vertebrates, testicular apoptosis is thought to be controlled by the pituitary gonadotropins Fsh and luteinising hormone (Lh). In fact, adult Atlantic bluefin tuna *Thunnus thynnus* [16] and greater amberjack *Seriola dumerili* [17,18] showed high levels of testicular apoptosis under captivity-induced reproductive dysfunction, a condition associated with reduced pituitary gonadotropin secretion [19]. In Atlantic cod, *Gadus morhua*, exposure to continuous light induced an increase in apoptotic germ cells (particularly proliferating spermatogonia), resulting in reduced androgen levels, a condition associated with insufficient gonadotropic stimulation [20].

Our objective was to gain further insight into the effects of rFsh administration in meagre spermatogenesis and to improve the existing knowledge on the role of apoptosis in spermatogenesis. Therefore, germ cell composition and testicular apoptosis were compared in the testes of rFsh-treated pre-pubertal meagre versus untreated controls and in adult meagre sampled in two phases of spontaneous spermatogenesis.

## 2. Materials and Methods

### 2.1. Sampling

This study was prompted by our previous studies on the effects of rFsh on pre-pubertal meagre spermatogenesis [9] and on reproductive maturation of adult meagre reared in commercial sea cages [21]. However, in the present study, a different set of analyses was performed in order to compare the spermatogenesis process induced by the rFsh treatment in pre-pubertal fish and the physiological process of spermatogenesis occurring in adults.

The pre-pubertal fish (*n* = 13) were 18-month-old individuals that belonged to a stock used for an experiment aimed at inducing precocious puberty through the administration of rFsh [9]. These fish (PreP-Fsh; *n* = 4) were reared in indoor tanks at IRTA (La Ràpita, Spain) and, to mimic the natural pattern of gonadotropin secretion, were administered increasing doses of rFsh for six weeks (week 0: 6 µg/kg; week 1: 9 µg/kg; week 2 to week 6: 12 µg/kg, which equated to approx. 0.45–0.9 mL). All injections were intramuscular, with volumes of 250 μL or less administered at various points in the dorsal muscle. Pre-pubertal control fish (PreP-C; *n* = 9) were injected weekly with 1 mL of saline solution. Detailed information on rFsh production, fish husbandry and experimental treatment is reported in [9]. The adult fish (*n* = 9) belonged to a commercial stock reared for six years at the fish farm Rehomare InMare S.r.l. in a sea cage off Gallipoli (Gulf of Taranto, Italy). These fish were sampled in late March-April 2021 (*n* = 5) and June 2021 (*n* = 4), which encompasses the reproductive season of this species in the Mediterranean [22]. According to the prevalent germ cell types present in the gonads, fish sampled in March-April were considered to be in the proliferative phase of spermatogenesis (group name: Adult-Pro), whereas fish sampled in June were considered to be in the meiotic phase (group name: Adult-Meio).

From each fish, biometric data (total length, TL, in cm; total body mass, BM, in g; gonad mass, GM, in g) were recorded (Table 1); then, one-centimetre-thick cross-sections were taken from the testes of each fish and fixed in Bouin’s solution for histological and apoptosis analysis.

### 2.2. Histological Analysis and Identification of Apoptotic Germ Cells

All fixed testis samples were embedded in paraffin wax. Subsequently, for each embedded sample, at least 20 deparaffinised sections, four micrometres thick, were stained with haematoxylin–eosin (H&E), and two sections were destined to the identification of apoptotic germ cells through the terminal deoxynucleotidyl transferase-mediated d’UTP nick end labelling (TUNEL) method with an in situ Cell Death Detection Kit, AP (Roche Diagnostics, Mannheim, Germany) [16,17]. Briefly, testis sections previously treated with a permeabilisation solution (0.1% Triton X-100 in 0.1% sodium citrate) for 8 min were incubated with the reaction mixture. The terminal deoxynucleotidyl transferase was diluted 1:2 in TUNEL Dilution Buffer (Roche Diagnostics, Mannheim, Germany), and a ready-to-use solution of nitro-blue tetrazolium chloride/5-bromo-4-chloro-3′-indolyphosphate p-toluidine salt (NBT/BCIP) (Roche Diagnostics, Mannheim, Germany) was used as a substrate for the signal conversion.

### 2.3. Relative Quantification of Germ Cell Types and Testicular Apoptosis

The histological analysis was carried out by estimating the density of the different germ cell stages in the germinal epithelium of five H&E-stained sections.

Prior to quantification, the germ cell types were identified on H&E-stained sections following the description reported in [23]. Briefly, the largest germ cells were the single undifferentiated type A (A_und_) spermatogonia (mean diameter 15.5 ± 1.8 μm); spermatogonia committed towards spermatogenesis (differentiated type A, A_diff_, and type B) were smaller cells contained in spermatocysts of two or more cells; spermatocyte morphology varied according to the different meiotic phases; and post-meiotic germ cells (spermatids and spermatozoa) were characterised by a compact and strongly basophilic nucleus.

The density of single type A_und_ spermatogonia (*n* cells/mm^2^ germinal epithelium) and the relative surface occupied by committed spermatogonia (type A_diff_ + type B), spermatocytes, and post-meiotic germ cells (germ cell cyst surface/mm^2^ of germinal epithelium) were measured in five randomly selected digital fields from the peripheral (proliferative) testis region and compared among groups (PreP-C, PreP-Fsh, Adult-Pro, and Adult-Meio). Spermatozoa released into the lumina of the seminiferous tubules after cyst opening were not included in the quantification.

To compare already published testicular apoptosis data of pre-pubertal meagre [9] with apoptosis data of adults undergoing spontaneous spermatogenesis, the surface area occupied by TUNEL-positive apoptotic structures (μm^2^/mm^2^ testis tissue) was measured in five randomly selected digital fields of Adult-Pro and Adult-Meio. All the above measurements were taken from microphotographs captured with a digital camera (K3, Leica, Wetzlar, Germany) connected to a light microscope (DMRB, Leica, Wetzlar, Germany) with a 40× objective, using an image-analysis software (Leica Application Suite X, version 5.1.0.25446, Wetzlar, Germany).

### 2.4. Statistical Analysis

Differences in the density of single type A_und_ spermatogonia, committed spermatogonia, spermatocytes, and post-meiotic germ cell cysts were evaluated by an ANOVA followed by Tukey–Kramer post hoc test. Statistical differences in apoptosis density data between the two adult meagre groups were assessed with Student’s *t*-test. Prior to the statistical analysis, normality of variance was tested through the Shapiro–Wilk W test, and percentage and proportion data were arcsine transformed [24]. A mixed-effects model, using Restricted Maximum Likelihood (REML), was applied to confirm that intra-specimen variability (random variance) was lower than inter-fish (group-level) variability across all parameters. Statistical analyses were performed using SAS^®^ OnDemand for Academics (SAS Institute Inc., Cary, NC, USA), and results were presented as means ± sd, with statistical significance set at *p* < 0.05.

## 3. Results

**Figure 1:**
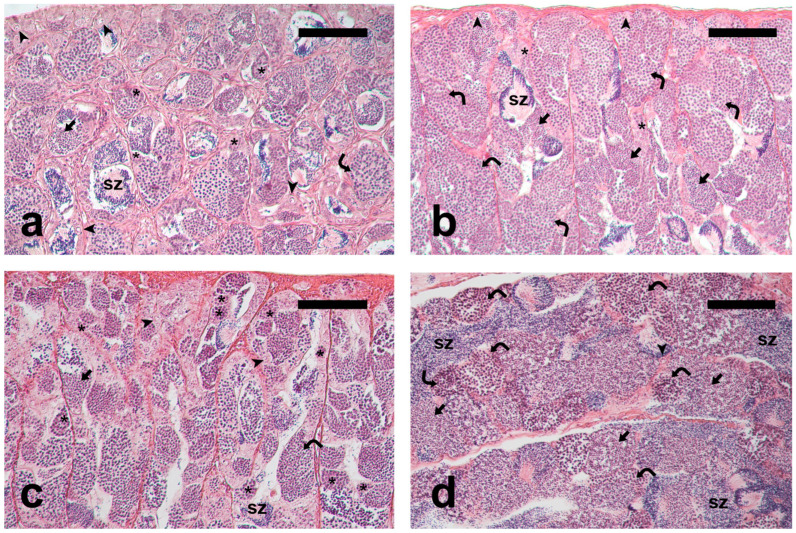
Micrographs of meagre testis sections. (**a**) Pre-pubertal control fish, PreP-C; (**b**) rFsh-treated pre-pubertal fish, PreP-Fsh; (**c**) Adult fish sampled in March-April, Adult-Pro; (**d**) Adult sampled in June, Adult-Meio. The presence of larger spermatocysts containing meiotic and post-meiotic cells indicates that PreP-Fsh and Adult-Meio were at a more advanced spermatogenic stage compared to PreP-C and Adult-Pro, respectively. Haematoxylin-eosin staining. Magnification bar = 100 μm. Arrowhead: single undifferentiated type A spermatogonium; asterisk: committed (differentiated type A and type B) spermatogonia; curved arrow: spermatocytes; arrow: post-meiotic germ cells; sz: spermatozoa.

PreP-C fish had small testes containing germ cells at all spermatogenic stages (Figure 1a); PreP-Fsh fish had larger testes and more active spermatogenesis compared to the PreP-C fish (Figure 1b). Adult-Pro had active testes showing all the germ cell types in the germinal epithelium and a limited number of luminal spermatozoa (Figure 1c). Adult-Meio had larger testes containing cysts with meiotic cells, as well as abundant post-meiotic cysts and luminal spermatozoa (Figure 1d), indicating a more advanced stage of spermatogenesis compared to the earlier group (Adult-Pro).

**Figure 2:**
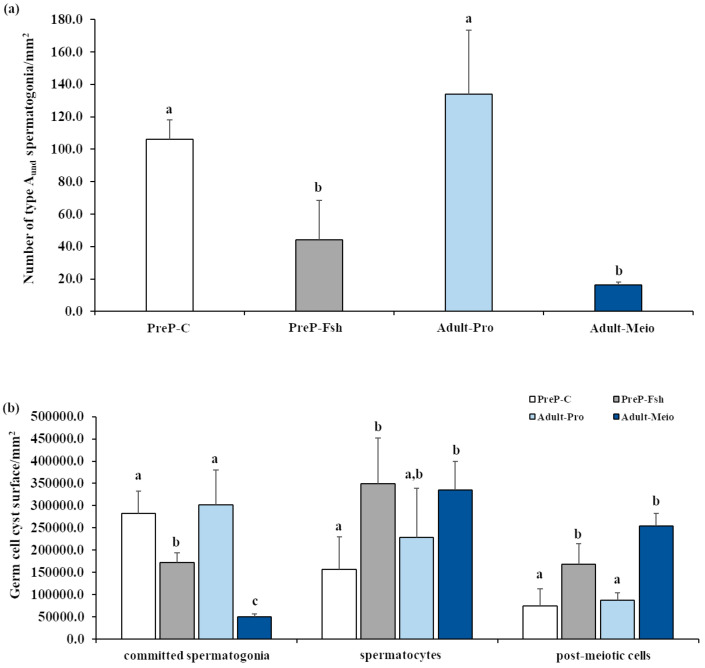
Relative quantification of germ cell types in testis sections of pre-pubertal control (PreP-C) and rFsh-treated (PreP-Fsh) fish and adult meagre in proliferative (Adult-Pro) and meiotic (Adult-Meio) stages of spermatogenesis. (**a**) Density of single type A undifferentiated spermatogonia (type A_und_); (**b**) Germinal epithelium surface occupied by committed spermatogonia (differentiated type A and type B), spermatocytes and post-meiotic (spermatids/spermatozoa) cells. Different letters represent statistically significant differences (ANOVA; *p* < 0.05).

The results of the relative quantification of germ cell types on testis sections are shown in Figure 2. The density of single type A_und_ spermatogonia in PreP-C was similar to that of Adults-Pro (Figure 2a). After the treatment with rFsh, the density of single type A_und_ decreased significantly (*p* < 0.05) and became similar to that of Adults-Meio (*p* = 0.33) (Figure 2a). Committed spermatogonia were the prevalent germ cell types in the testes of PreP-C fish and in Adult-Pro fish; hence, the testes of both these fish groups were in the proliferative phase of spermatogenesis. A statistically significantly lower density of committed spermatogonia was observed in PreP-Fsh compared with PreP-C and in Adult-Meio compared with Adult-Pro (*p* < 0.05 for both comparisons) (Figure 2b). In both comparisons, the lower density of committed spermatogonia was associated with an increase in both meiotic and post-meiotic germ cells (*p* < 0.05 for both comparisons) (Figure 2b). Spermatocytes were the prevalent germ cell types in PreP-Fsh and Adult-Meio; hence, these groups of fish were considered to be in the meiotic phase of spermatogenesis.

**Figure 3:**
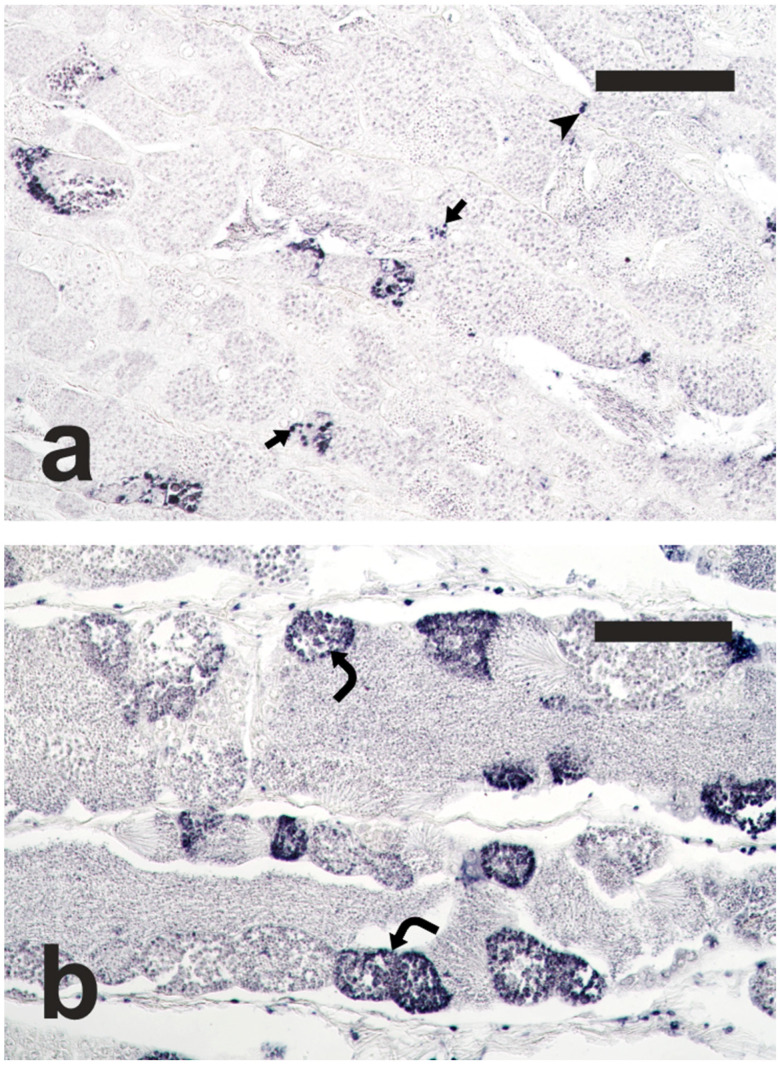
Micrographs of adult meagre testis sections stained with the terminal deoxynucleotidyl transferase-mediated 2′-deoxyuridine 5′-triphosphate nick end labelling (TUNEL) method, showing nuclei of apoptotic germ cells stained in dark blue. (**a**) Adult fish in the proliferative phase of spermatogenesis, Adult-Pro; (**b**) Adult fish in the meiotic phase of spermatogenesis, Adult-Meio. Magnification bars = 100 μm. Arrow: TUNEL-positive Sertoli cell; arrowhead: TUNEL-positive spermatogonia; curved arrow: TUNEL-positive spermatocytes.

The TUNEL staining, performed on testis sections of adult meagre, labelled apoptotic cells, including spermatogonia, spermatocytes and Sertoli cells (Figure 3). In the Adult-Meio group, a high number of cysts containing apoptotic germ cells was observed, and apoptosis often involved all the germ cells contained in the affected cysts. The surface area of apoptotic structures was significantly greater in the Adult-Meio group than in the Adult-Pro group (32,147.3 ± 18,796.2 µm^2^/mm^2^ vs. 11,082.4 ± 4925.5 µm^2^/mm^2^; Student’s t-test; *p* < 0.05), indicating that the spermatogenesis progression was associated with an increase in apoptosis.

## 4. Discussion

The quantification of the different germ cell types performed in the present study confirmed our previous observations based on the analysis of germ cell proliferation [9]. Although based on a limited number of specimens, the study by Zupa et al. [9] showed that administration of rFsh for six weeks effectively triggered the activation of Fsh/Lh receptors and steroidogenesis in 18-month-old pre-pubertal meagre. These fish showed signs of spermatogenesis activation but were unable to finalise the first reproductive cycle and did not produce releasable sperm, presumably due to insufficient endogenous Lh release from the pituitary. In the present study, we showed that the treatment clearly advanced spermatogenesis, as spermatocytes became the predominant germ cell type in the testes of rFsh-treated pre-pubertal meagre, resembling the pattern observed in adult fish at an advanced stage of spermatogenesis.

In vertebrates, germ cell apoptosis is an important homeostatic process that occurs during normal testicular function [13,25] and has been documented in all three phases of spermatogenesis: proliferative or spermatogonial, meiotic or spermatocytary, and spermiogenesis or differentiation [13,26,27,28,29,30,31,32,33]. The rate of apoptosis of committed spermatogonia is thought to be responsible for the spermatogenesis efficiency, a species-specific reproductive trait that determines the final sperm output [32,34,35,36]. Gonadotropins, particularly Fsh, and androgens such as testosterone (T) act as survival factors for germ cells. Withdrawal of Fsh and T has been correlated with increased germ cell apoptosis across different vertebrate classes [13,15,16,17,20,37,38,39,40,41,42,43,44,45]. The existence of an inverse correlation between male germ cell proliferation and apoptosis, with an increase in apoptosis during the testicular regression/non-spermatogenic phase (corresponding to low androgen levels), has been reported in mammals [13,46,47,48], birds [49,50], reptiles [51], and amphibians [52,53].

In amniotes, Sertoli cells do not proliferate, and apoptosis is involved in maintaining the correct germ cell/Sertoli cell ratio throughout the spermatogenic process [13,35,54,55,56,57,58], a function that adds to the general role of apoptosis in preventing the maturation of aberrant germ cells [13,54]. In the cystic testis of anamniotes, however, Sertoli cells divide in parallel with spermatogonial proliferation [15,20,59,60,61]; therefore, a role of apoptosis in maintaining the optimal germ cell/Sertoli cell ratio is implausible, and its biological function needs to be further elucidated, as variations in this process can occur depending on the species, reproductive phase, environmental conditions and ageing [28,31,62,63,64].

In adult teleost fish with annual reproductive cycles, such as swordfish *Xiphias gladius* [62], Atlantic cod [32], Atlantic bluefin tuna [16], the Lake Van fish *Chalcalburnus tarichi* [65], and greater amberjack [17], higher rates of testicular apoptosis have been reported coinciding with the maximum germ cell proliferation activity and the highest plasma levels of Fsh/Lh and androgens. This finding aligns with the hypothesised role of germ cell apoptosis in eliminating aberrant cells and is consistent with the results of our study, where we observed a high density of apoptotic cells during the meiotic phase, coinciding with elevated plasma concentrations of 11-KT [21]. However, in teleost fishes, as in seasonally breeding mammals, Fsh/Lh and sex steroids act as survival factors for germ cells. Indeed, several genes involved in the initiation of germ cell apoptosis, whose expression correlates with Fsh levels, have been recently identified [66,67]. In Atlantic cod, the inhibition of the reproductive axis through exposure to a long photoperiod, which is associated with insufficient stimulation, resulted in an increase in apoptosis of late spermatogonia and Sertoli cells, and the restoration of the normal photoperiod reduced apoptosis levels [20]. In zebrafish, Fsh stimulated retinoic acid production, which promoted spermatogonia differentiation, supported 11-KT-stimulated meiosis, and reduced germ cell apoptosis [68]. At the start of rFsh treatment, pre-pubertal meagre (PreP-C group) exhibited high levels of testicular apoptosis (23,451.4 ± 1554.8 µm^2^/mm^2^), predominantly affecting spermatogonia and Sertoli cells [9]. However, following rFsh treatment (PreP-Fsh group), apoptosis decreased markedly (1700.8 ± 297.3 µm^2^/mm^2^) [9], reaching levels similar to or even below those observed in adults (present study). The evidence that germ cell apoptosis peaks concomitantly with the peak of spermatogenesis and the reported role of Fsh/Lh and sex steroids as germ cell survival factors are in apparent reciprocal contradiction. By combining the data collected in this study—albeit limited by the absence of a complete steroid dataset—with findings from the relevant literature, a general pattern of apoptosis during spermatogenesis in teleost fishes exhibiting seasonal reproductive cycles emerged. During the proliferative phase of spermatogenesis (or spermatogonial phase), characterised by rapid spermatogonial proliferation, the increasing levels of androgens exert their protective action against germ cell death, and apoptosis is very low. At this stage, apoptosis mainly affects individual spermatogonia within the spermatocysts; the rate of apoptosis is species-specific and is responsible for the efficiency of spermatogenesis. However, in pre-pubertal fish that attempt the first reproductive cycle, apoptosis is very high due to the insufficient Fsh/Lh and sex steroid levels, and spermatogonia that cannot proceed towards spermatogenesis die. In this condition, spermatogenesis efficiency is negligible, and releasable spermatozoa are not produced. In adults, when spermatogenic activity is at its peak and spermatocytes are the predominant cell types in the seminiferous tubules (meiotic phase), apoptosis is high and no longer correlates with sex hormones, suggesting that apoptosis in this phase decouples from androgens. Finally, coherently with literature data on sand steenbras *Lithognathus mormyrus* [69], gilthead seabream *Sparus aurata* [28,70], swordfish [62] and greater amberjack [17], when androgens withdraw, 17β-estradiol increases and testes prepare for a quiescent stage at the end of the reproductive season; residual germ cells that cannot proceed in the spermatogenic process are removed by apoptosis. This has also been observed in adult post-spawning meagre (R.Z., unpublished data). Not only does the role of apoptosis appear to change during the spermatogenic process, but the way in which apoptosis occurs also changes. In fact, in pre-pubertal meagre [9] and in adults sampled during the proliferation phase (present study), apoptosis affected mainly one or a few individual spermatogonia within a spermatocyst, implying that the cell death mechanism must involve the isolation of the spermatogonia undergoing apoptosis from other cells of the spermatocyst clone, as already observed in cod [32]. Incidentally, in vitro experiments on a mammal epithelial cell line indicated that detachment from neighbouring cells, substratum, or extracellular matrix is likely to be a specific component of the apoptotic process [71]. On the contrary, during the meiotic and post-meiotic phases, apoptotic cells may not need to be isolated because apoptosis often affects the entire germ cell clone of a spermatocyst.

## 5. Conclusions

Although constrained by the absence of comprehensive steroid hormone profiles and a limited sample size, this study corroborates our previous observations on the effects of Fsh on pre-pubertal meagre testes and offers new insights into the dynamic role of apoptosis during the pre-pubertal phase and throughout spermatogenesis. Future research with larger, hormone-characterised samples across teleost species with varied reproductive strategies is warranted to validate the proposed apoptotic pattern.

## Figures and Tables

**Table 1:** Mean (±sd) total length, body mass, and gonad mass in pre-pubertal and adult meagre.

Fish Group	Total Length (cm)	Body Mass (g)	Gonad Mass (g)
Pre-pubertal—control (PreP-C; *n* = 9) *	49.2 ± 4.3	1125.1 ± 266.3	1.5 ± 0.8
Pre-pubertal—rFsh-treated (PreP-Fsh; *n* = 4) *	46.0 ± 3.2	906.5 ± 172.1	7.7 ± 2.6
Adults—March-April (Adult-Pro; *n* = 5)	71.8 ± 3.0	3396.0 ± 157.3	28.3 ± 16.3
Adults—June (Adult-Meio; *n* = 4)	72.4 ± 2.1	3512.5 ± 375.0	142.3 ± 35.0

* Pre-pubertal fish belonged to a stock used for an experiment aimed at inducing precocious puberty through the administration of rFsh [9].

## Data Availability

The original contributions presented in this study are included in the article. Further inquiries can be directed to the corresponding author.
